# Novelty triggers time-dependent theta oscillatory dynamics in cortical-hippocampal-midbrain circuitry

**DOI:** 10.1186/s13041-024-01167-6

**Published:** 2024-12-18

**Authors:** Alan Jung Park

**Affiliations:** 1 Department of Physiology, Seoul National University College of Medicine, Seoul, Republic of Korea; 2 Neuroscience Research Institute, Seoul National University College of Medicine, Seoul, Republic of Korea; 3 Department of Biomedical Sciences, Seoul National University College of Medicine, Seoul, South Korea; 4 Wide River Institute of Immunology, Seoul National University, Seoul, South Korea; 5 The Mortimer B. Zuckerman Mind Brain Behavior Institute at Columbia University, New York, NY 10032 USA

**Keywords:** Novelty, Medial prefrontal cortex, Ventral hippocampus, Ventral tegmental area, Dopamine D1 receptor, Theta

## Abstract

Rapid adaptation to novel environments is crucial for survival, and this ability is impaired in many neuropsychiatric disorders. Understanding neural adaptation to novelty exposure therefore has therapeutic implications. Here, I found that novelty induces time-dependent theta (4-12Hz) oscillatory dynamics in brain circuits including the medial prefrontal cortex (mPFC), ventral hippocampus (vHPC), and ventral tegmental area (VTA), but not dorsal hippocampus (dHPC), as mice adapt to a novel environment. Local field potential (LFP) recordings were performed while mice were freely behaving in a novel or a familiar arena for 10 min. Initially, mice exhibited increased exploratory behavior upon exposure to novelty, which gradually decreased to levels observed in mice exposed to the familiar arena. Over the same time course, the mPFC, vHPC, and VTA displayed progressively increasing theta power through novelty exposure. Additionally, theta coherence and theta phase synchrony measures demonstrated that novelty weakened the connectivity between these areas, which then gradually strengthened to the level observed in the familiar group. Conversely, mice exposed to the familiar arena showed steady and consistent behavior as well as theta dynamics in all areas. Treatment with a dopamine D1-receptor (D1R) antagonist in the vHPC disrupted neurophysiological adaptation to novelty specifically in the vHPC-mPFC and vHPC-VTA circuits, without affecting behavior. Thus, novelty induces distinct theta dynamics that are not readily dictated by behavior in the mPFC, vHPC, and VTA circuits, a process mediated by D1Rs in the vHPC. The observed time-dependent circuit dynamics in the key learning and memory circuit would provide new insights for treating neuropsychiatric disorders that often show impaired novelty processing.

**Supplementary Information:**

The online version contains supplementary material available at 10.1186/s13041-024-01167-6.

## Introduction

Exploration and subsequent adaptation to a novel environment are fundamental for survival. This process necessitates the active processing of newly acquired information, requiring coordinated activity across brain circuitry. Indeed, novelty recruits distinct brain regions including the mPFC, HPC, and VTA, the major dopamine source [1–5]. Previous studies reported that exposure to a novel environment subsequently facilitates learning and memory retention by modulating communication between these areas [6–9]. However, it is unknown how the mPFC, HPC, and VTA interact over the course of spatial navigation in a novel environment. Because many neurodevelopmental and neuropsychiatric disorders such as autism and schizophrenia are associated with maladaptive behavior upon novelty exposure [10, 11], deciphering time-dependent changes in neural circuit dynamics that occur upon novelty exposure can help treat cognitive impairments in these conditions.

Theta frequency oscillations are prominent during active navigation and support various cognitive processes including memory and stimuli sampling. Moreover, they coordinate inter-regional brain activities for effective information processing [12]. Indeed, exposure to a novel environment increases overall theta power in the mPFC and vHPC, with the latter remaining persistent at least one hour after novelty exposure [8]. Theta coherence in the vHPC-mPFC circuit increases as a function of learning, indicating that theta synchrony in this circuit represents memory encoding [13]. However, theta oscillatory dynamics in the vHPC-mPFC circuit over the course of novelty exposure have yet to be investigated.

VTA dopamine neurons respond to novelty and mediate enhanced learning following novelty exposure via their direct projection to the mPFC and vHPC [5, 8, 14]. Dopamine D1Rs gate vHPC-mPFC connectivity, and D1Rs within the vHPC mediate novelty-enhanced theta power in this area [8, 15, 16]. Moreover, antagonizing D1Rs in the vHPC impairs enhanced learning following novelty by blocking learning-dependent increases in theta coherence in the vHPC-mPFC, and vHPC-VTA circuits [8, 9]. These findings suggest that D1Rs in the vHPC are critical mediators through which novelty facilitates learning. Notably, the role of D1Rs during novelty exposure is unknown, and therefore investigating how D1Rs in the vHPC mediate theta oscillatory dynamics among the vHPC, mPFC, and VTA during novelty exposure is a critical next step.

I hypothesized that theta oscillatory activities in vHPC-mPFC-VTA circuitry represent coordinated time-dependent dynamics as initial saliency subsides over the course of novelty exploration, a process mediated by D1Rs in the vHPC. To test this, I performed simultaneous local field potential recordings in the vHPC, dHPC, mPFC, and VTA while mice explored a novel or a familiar environment. Analyzing theta power and theta synchrony revealed that novelty tightly orchestrates the interaction of the vHPC with the mPFC, and VTA, but not dHPC, via a mechanism that requires D1Rs in the vHPC. While previous research has primarily assessed general impacts of novelty on specific local circuits [8], this study is the first to reveal temporally changing interactions within the mPFC-HPC-VTA circuit over the course of novelty exposure, highlighting adaptive neurophysiological responses to novelty.

## Results

### Novelty exposure increases initial exploratory behavior

**Fig. 1:**
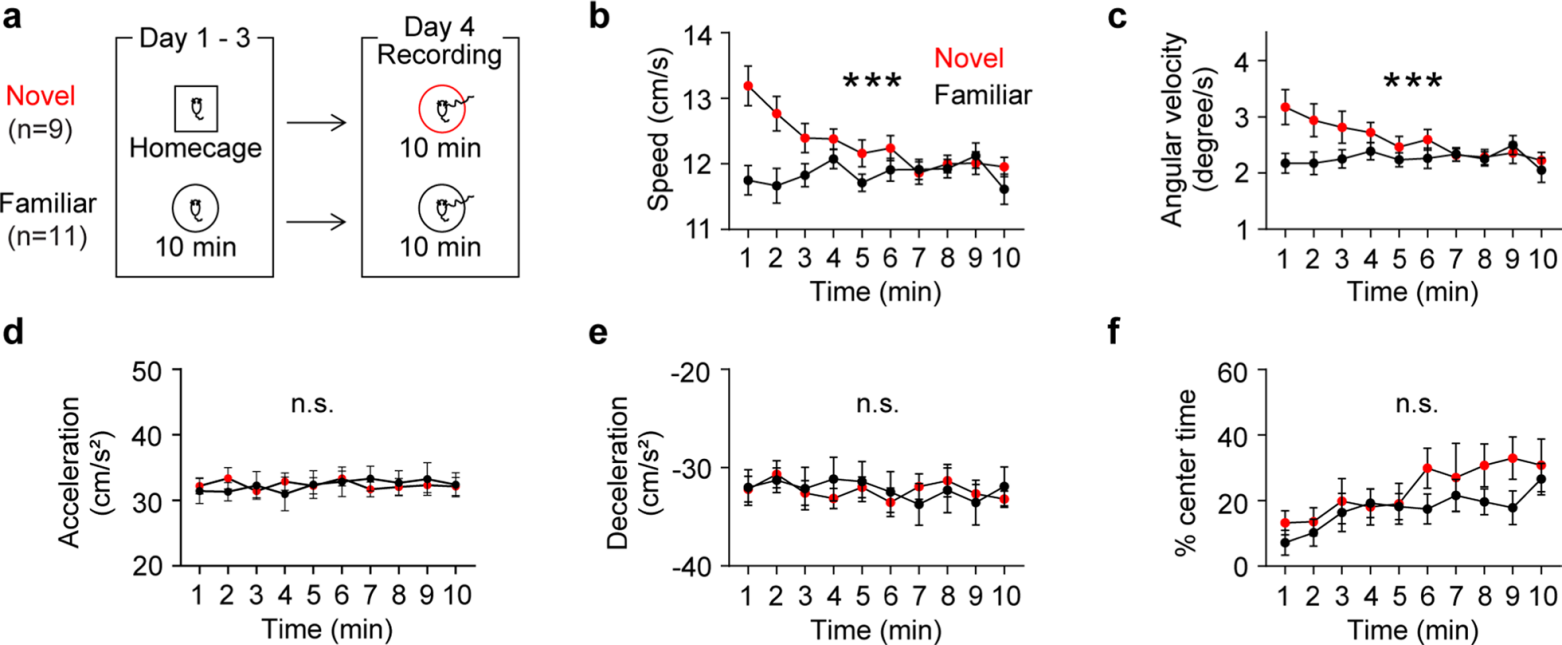
Novel arena exposure entails time-dependent behavioral changes in specific domains. **a** Experimental design. **b** Mice exposed to a novel arena exhibited a higher initial speed that decreased over time, whereas mice exposed to a familiar arena maintained a consistent speed throughout the exposure (time x group, P < 0.0001; time, P = 0.03; group, P = 0.04). **c** The novel group displayed a higher initial angular velocity that decreased over time, while the familiar group showed stable angular velocity throughout arena exposure (time x group, P = 0.0001; time, P = 0.05; group, P = 0.1). **d**-**f** Both groups of mice exhibited similar acceleration (**d**: time x group, P = 0.4; time, P = 0.5; group, P = 0.9), deceleration (**e**: time x group, P = 0.5; time, P = 0.3; group, P = 0.9), and time spent in the center (**f**: time x group, P = 0.5; time, P = 0.01; group, P = 0.3). Two-way RM ANOVA test. N.S., not significant. ***P < 0.0005. Data are represented as mean ± SEM

To assess behavioral impacts of spatial novelty exposure, I examined changes in behavior across 10 min while mice explored a circular arena that they had never experienced before (novel group). As a control, a separate group of mice (familiar group) experienced the same circular arena for three days prior to the experimental day in order for the mice to be familiarized with the arena (Fig. 1a). Mice exposed to the novel arena exhibited higher moving speed (Fig. 1b) and angular velocity (Fig. 1c) at the beginning of the exposure, which dissipated over time. Conversely, mice exposed to the familiar arena showed consistent moving speed and angular velocity throughout the arena exposure (Fig. 1b-c). Because a recent study demonstrated that brain oscillatory activity is controlled by movement acceleration, but not speed [17], I measured both acceleration and deceleration during arena exposure. These measures were steady and comparable between the novel and familiar groups throughout arena exploration (Fig. 1d-e). Finally, I examined the ratio of time mice explored in the center of the arena, a well-established indicator of anxiety levels, and both groups of mice displayed gradual increases in time spent in the center during arena exploration (Fig. 1f). Overall, these findings demonstrate that exposure to novelty enhances initial exploratory behavior.

### Novelty selectively impacts oscillatory dynamics in specific circuits

**Fig. 2:**
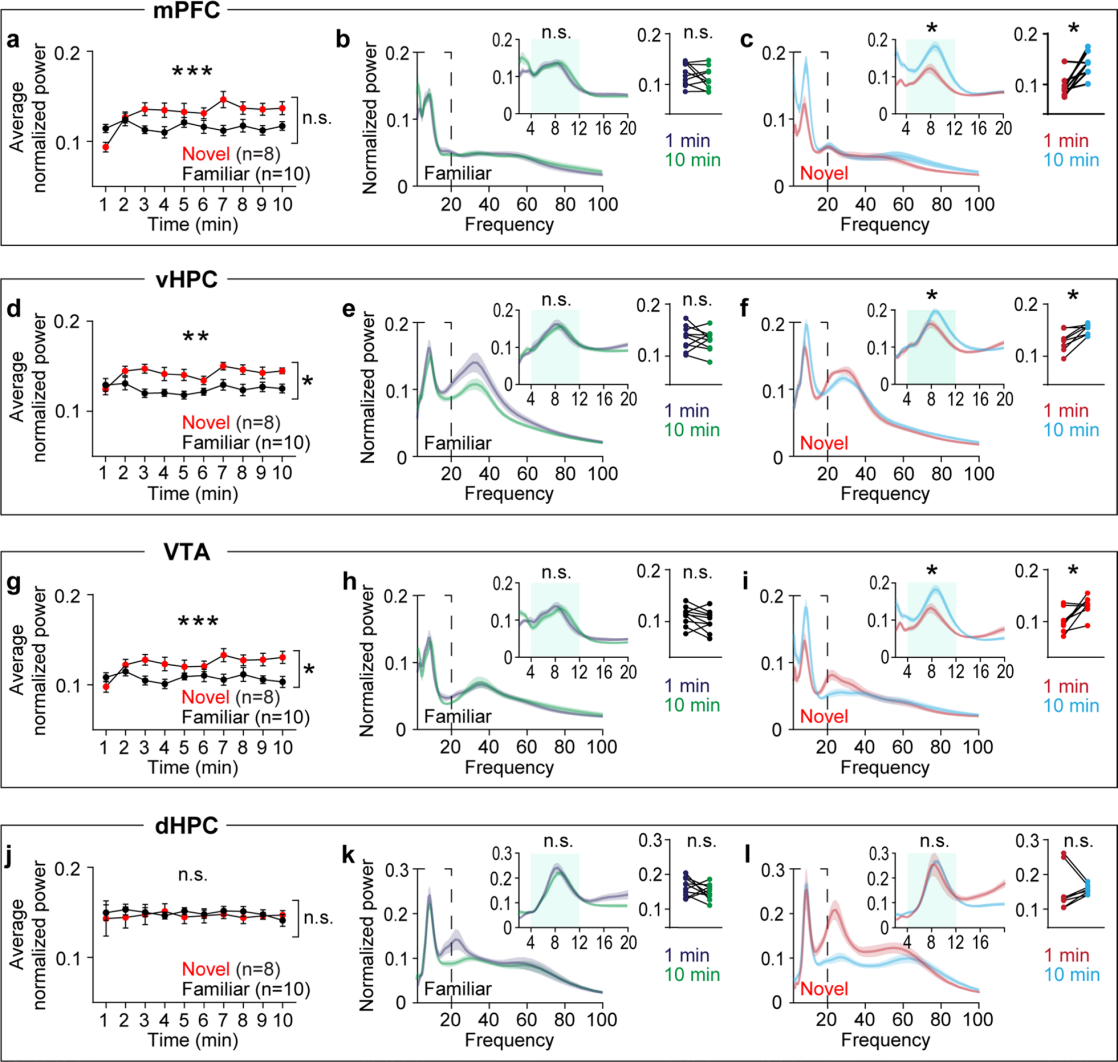
Novelty exposure gradually increases theta power in specific areas in the HPC-mPFC-VTA circuit. **a**-**c** In the mPFC, mice exposed to the novel, but not the familiar, area displayed progressive increases in theta power (**a**: time x group, P < 0.0001; time, P = 0.09; group, P = 0.08; **b**: P = 0.3; **c**: P = 0.008). **d**-**f** In the vHPC, the novel group displayed progressive increases in theta power compared with the familiar group (**d**: time x group, P = 0.003; time, P = 0.08; group, P = 0.01; **e**: P = 0.8; **f**: P = 0.008). **g**-**i** In the VTA, the novel group showed gradual increases in theta power relative to the familiar group (**g**: time x group, P = 0.0002; time, P = 0.07; group, P = 0.03; **h**: P = 0.3; **i**: P = 0.02). **j**-**l** In the dHPC, both groups displayed similar theta power during arena exposure (**j**: time x group, P = 0.9; time, P = 0.8; group, P = 0.7; **k**: P = 0.6; **l**: P = 0.8). Inset: Left, magnification of the area enclosed by dashed lines. Right, average normalized power in the shaded area. Two-way RM ANOVA for (**a**, **d**, **g**, **j**). Wilcoxon signed-rank test for the rest. N.S., not significant. *P < 0.05, **P < 0.005, ***P < 0.0005. Data are represented as mean ± SEM

To dissect the neurophysiological impact of novelty, I examined LFP changes during novelty exposure. I performed parametric two-way repeated measures (RM) ANOVA tests over 10 min of arena exposure followed by non-parametric tests to confirm significance. This cross validation revealed that novelty exposure selectively increases theta power in specific brain areas. Compared with the familiar group, the novel group showed progressive increases in theta power in the mPFC, vHPC, and VTA, with a pronounced group-level difference in the vHPC and VTA. However, the familiar group displayed consistent theta power throughout arena exposure (Fig. 2a-i). On the other hand, both groups of mice exhibited similar stable theta power over time in the dHPC (Fig. 2j-l). These effects are specific to theta frequency oscillations, as no significant time course differences were observed between the two groups in beta and gamma frequency oscillations (Supplemental Fig. 1 & 2).

**Fig. 3:**
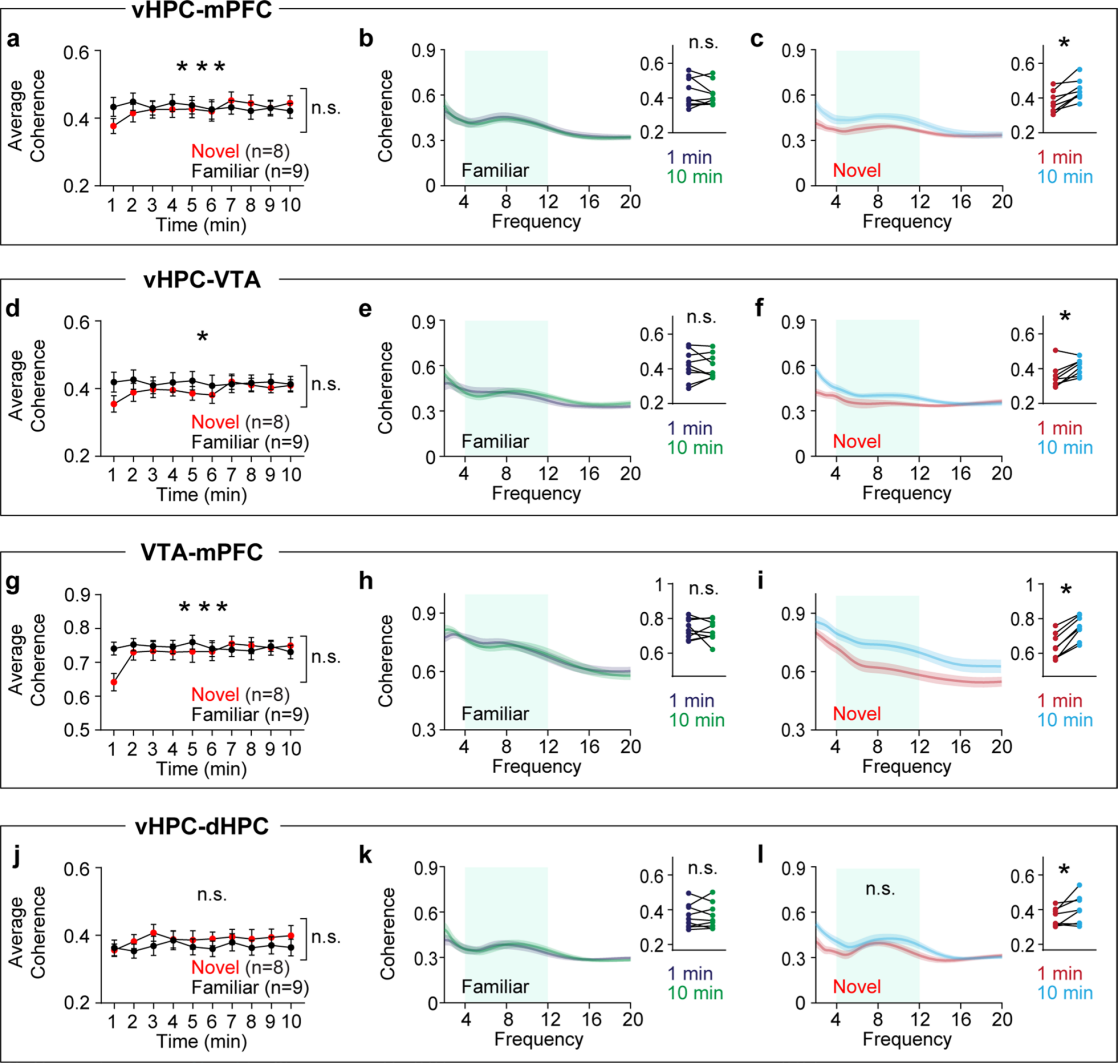
Novelty exposure impacts theta coherence dynamics in specific circuits between the HPC, mPFC, and VTA. **a**-**c** The novel, but not the familiar, group exhibited increasing vHPC-mPFC theta coherence during arena exposure (**a**: time x group, P < 0.0001; time, P = 0.1; group, P = 0.8; **b**: P = 0.7; **c**: P = 0.008). **d**-**f** The novel group displayed progressive increases in vHPC-VTA theta coherence compared with the familiar group (**d**: time x group, P = 0.008; time, P = 0.2; group, P = 0.5; **e**: P = 0.9; **f**: P = 0.02). **g**-**i** The novel group showed gradual increases in VTA-mPFC theta coherence relative to the familiar group (**g**: time x group, P < 0.0001; time, P = 0.02; group, P = 0.6; **h**: P = 0.9; **i**: P = 0.008). **j**-**l** In the dHPC, both groups displayed similar theta power during arena exposure (**j**: time x group, P = 0.9; time, P = 0.9; group, P = 0.5; **k**: P = 0.9). Within-group analysis between 1- and 10 min revealed increasing theta coherence in the novel group (**l**: P = 0.04). Inset: average normalized power in the shaded area. Two-way RM ANOVA for (**a**, **d**, **g**, **j**). Wilcoxon signed-rank test for the rest. N.S., not significant. *P < 0.05, ***P < 0.0005. Data are represented as mean ± SEM

**Fig. 4:**
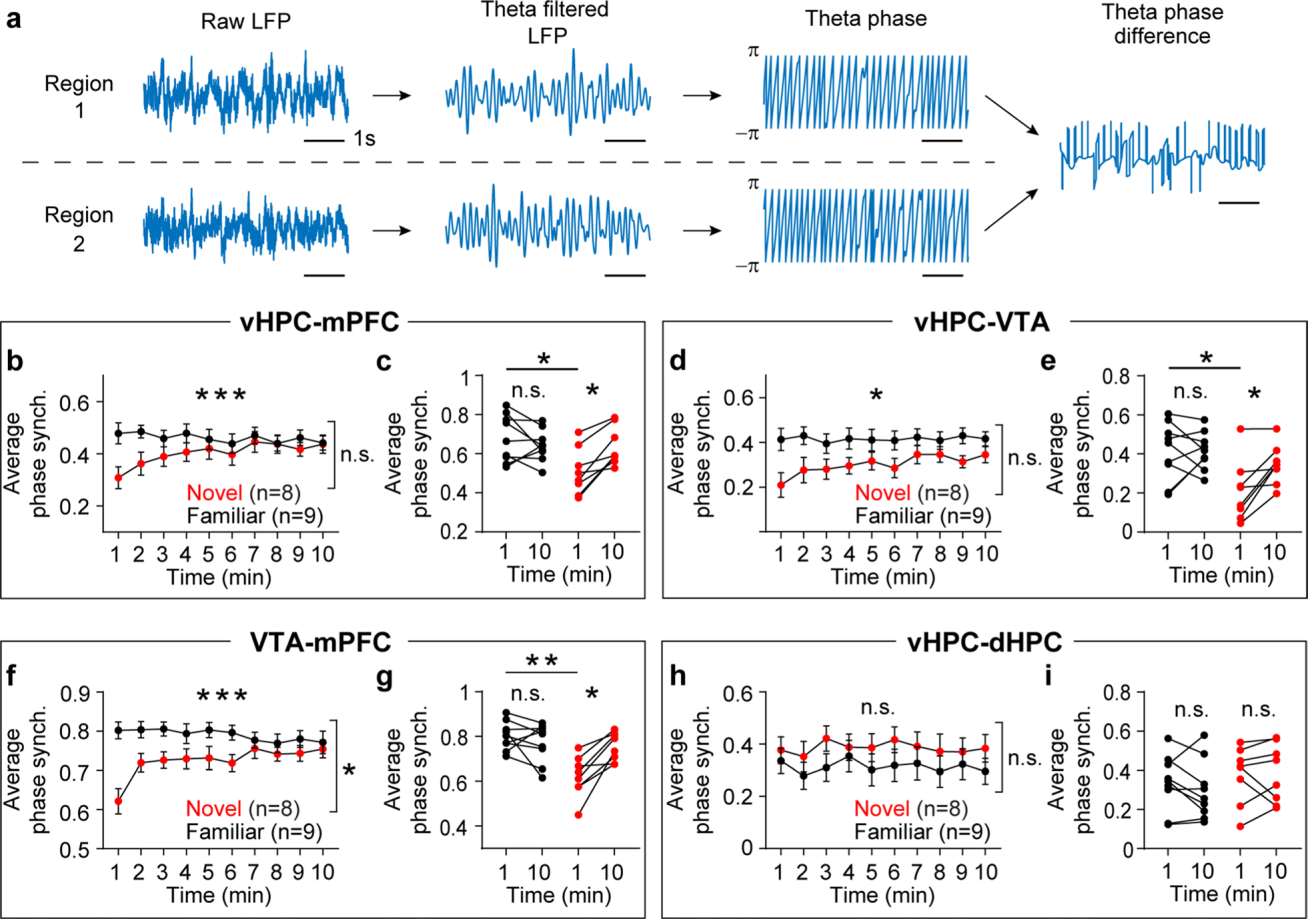
Novelty exposure impacts theta phase synchrony dynamics in specific circuits between the HPC, mPFC, and VTA. **a** Schematic of extracting theta phase angle differences to measure phase synchrony between two regions. **b-g** The novel group exhibited decreased theta phase synchrony in the vHPC-mPFC, vHPC-VTA, and VTA-mPFC circuits at 1 min, which gradually recovered during arena exposure compared with the familiar group (**b**: time x group, P < 0.0001; time, P = 0.1; group, P = 0.2; **c**: familiar vs. novel at 1 min: P = 0.03; familiar vs. novel at 10 min: P = 0.9; familiar 1- vs. 10min: P = 0.4; novel 1- vs. 10min: P = 0.008; **d**: time x group, P = 0.01; time, P = 0.1; group, P = 0.05; **e**: familiar vs. novel at 1 min: P = 0.03; familiar vs. novel at 10 min: P = 0.9; familiar 1- vs. 10min: P = 0.9; novel 1- vs. 10min: P = 0.008; **f**: time x group, P < 0.0001; time, P = 0.08; group, P = 0.03; **g**: familiar vs. novel at 1 min: P = 0.002; familiar vs. novel at 10 min: P = 0.9; familiar 1- vs. 10min: P = 0.4; novel 1- vs. 10min: P = 0.008). **h-i** Both groups of mice displayed similar vHPC-dHPC theta phase synchrony during arena exposure (**h**: time x group, P = 0.3; time, P = 0.2; group, P = 0.4; **i**: familiar vs. novel at 1 min: P = 0.9; familiar vs. novel at 10 min: P = 0.9; familiar 1- vs. 10min: P = 0.4; novel 1- vs. 10min: P = 0.7). Two-way RM ANOVA for (**b**, **d**, **f**, **h**). Kruskal–Wallis test followed by Dunn’s post-hoc test for between group analysis, and Wilcoxon signed-rank test for within group analysis. N.S., not significant. * P < 0.05, ** P < 0.005, *** P < 0.0005. Data are represented as mean ± SEM

I hypothesized that novelty would increase synchrony in theta frequency over time among the mPFC, vHPC, and VTA because all of these areas show increasing theta power during novelty exposure. To test this hypothesis, synchrony was measured using coherence [18]. Although RM ANOVA tests did not find group-level differences between the novel and familiar groups, within group analyses demonstrated that the novel group displayed gradual increases in theta coherence in vHPC-mPFC, vHPC-VTA, and VTA-mPFC circuitry (Fig. 3a-i). Both groups showed similar levels of theta coherence in the vHPC-dHPC circuit (Fig. 3j-l). Because coherence measures phase angle differences weighted by power [18], instantaneous phase angle differences can be masked by concurrent power changes in these areas. Thus, I measured synchrony based on theta phase angle differences (Fig. 4a). Consistent with coherence findings (Fig. 3), theta phase synchrony progressively increased over time in the vHPC-mPFC, vHPC-VTA, and VTA-mPFC circuits only in the novel group. Moreover, theta phase synchrony at the onset of arena exposure was significantly lower in the novel group compared with the familiar group in these circuits (Fig. 4b-g). Both groups of mice exhibited similar theta phase synchrony in the vHPC-dHPC circuit (Fig. 4h-i). Collectively, these findings indicate that novelty specifically increases theta power over time in the mPFC, vHPC, and VTA, an effect accompanied by plastic changes in theta synchrony between these areas.

### Dopamine D1-receptors in the vHPC mediate physiological effects of novelty

**Fig. 5:**
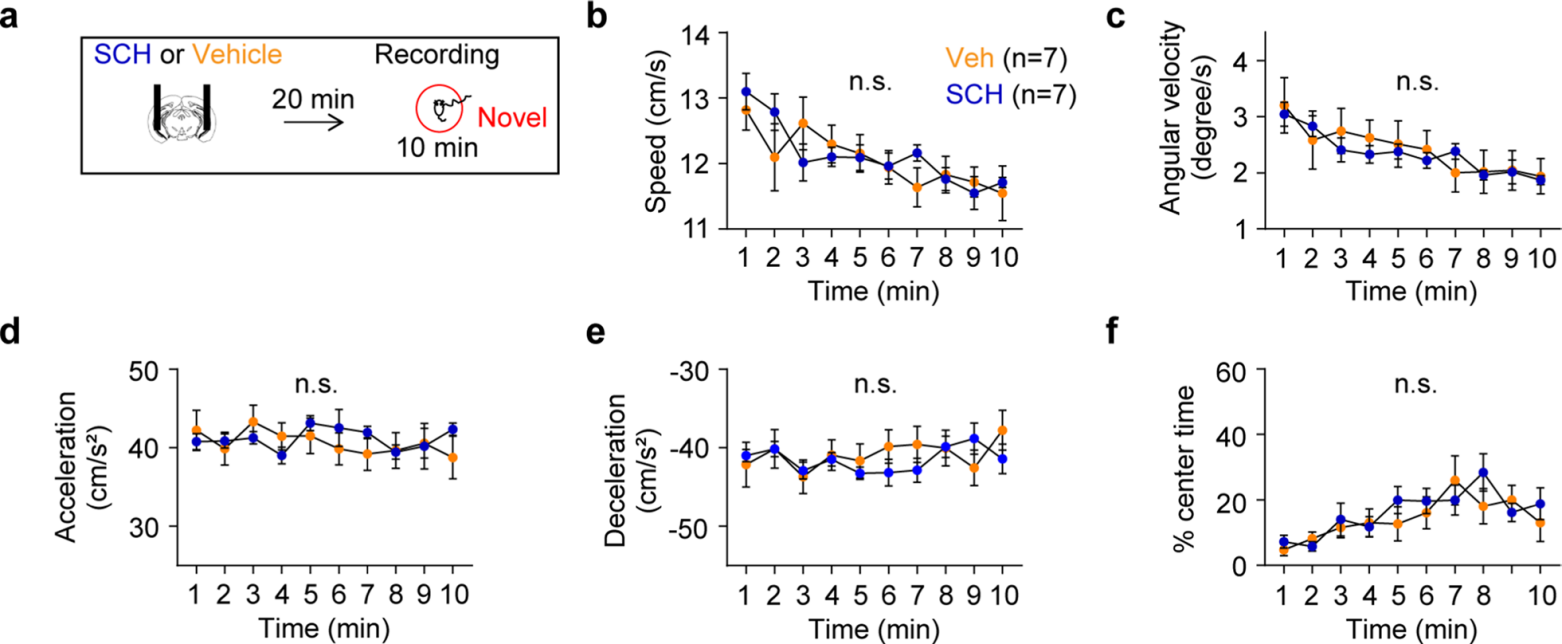
Antagonizing D1 receptors in the vHPC does not affect behavior during novel arena exposure. **a** Experimental design. **b-f** Mice infused with SCH or vehicle into the vHPC exhibited similar speed (**b**: time x group, P = 0.07; time, P = 0.001; group, P = 0.8), angular velocity (**c**: time x group, P = 0.5; time, P = 0.002; group, P = 0.2), acceleration (**d**: time x group, P = 0.2; time, P = 0.4; group, P = 0.8), deceleration (**e**: time x group, P = 0.07; time, P = 0.2; group, P = 0.8), and time spent in the center (**f**: time x group, P = 0.3; time, P = 0.004; group, P = 0.9). Two-way RM ANOVA test. N.S., not significant. Data are represented as mean ± SEM

**Fig. 6:**
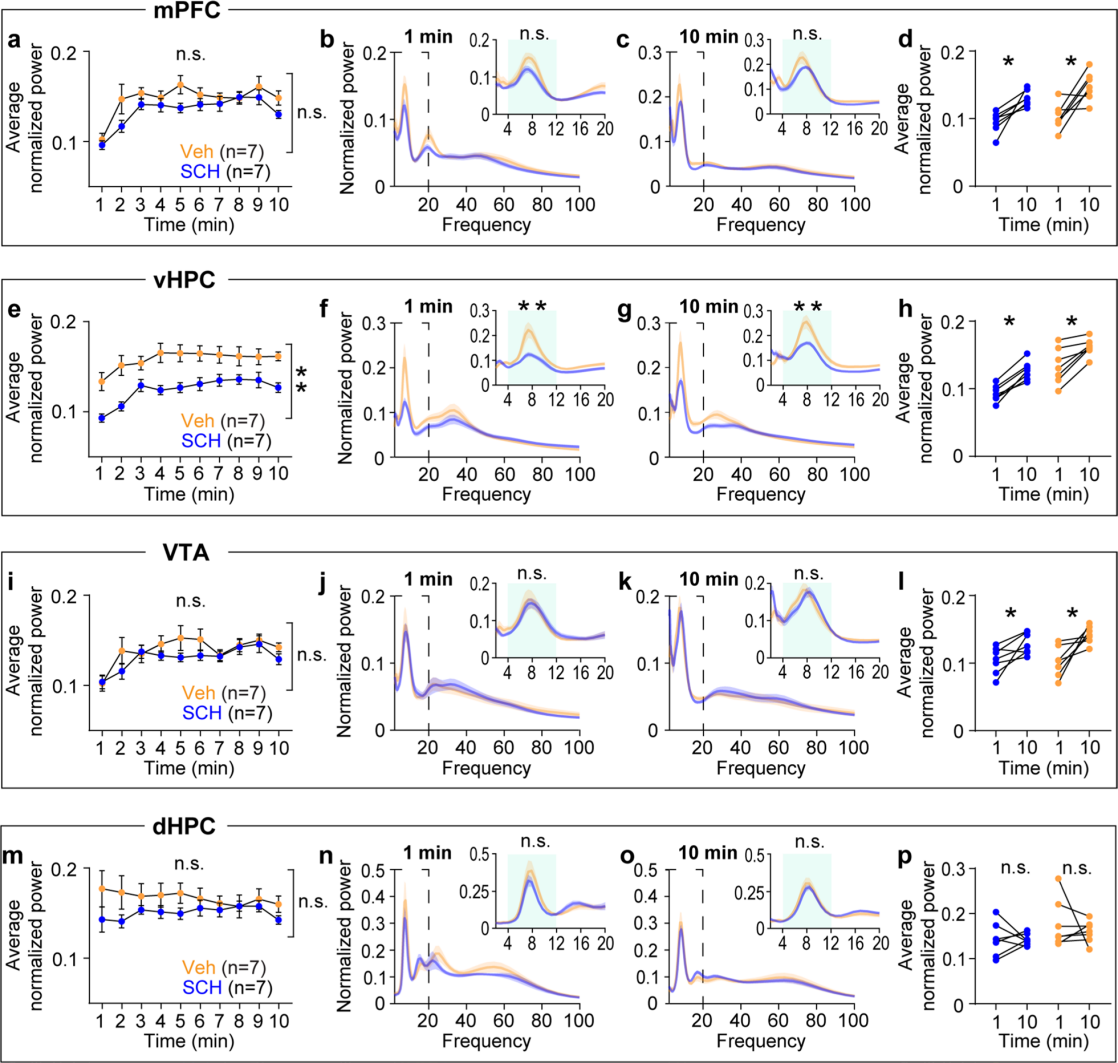
Antagonizing D1 receptors in the vHPC selectively reduces theta power in the vHPC during novel arena exposure. **a**-**d** In the mPFC, mice infused with SCH or vehicle into the vHPC exhibited similar theta power (**a**: time x group, P = 0.6; time, P < 0.0001; group, P = 0.05; **b**: P = 0.07; **c**: P = 0.1; **d**: SCH, P = 0.02, Veh, P = 0.04). **e**–**h** In the vHPC, the SCH group displayed decreased theta power compared with the Veh group (**e**: time x group, P = 0.2; time, P = 0.0003; group, P = 0.003; **f**: P = 0.004; **g**: P = 0.001; **h**: SCH, P = 0.02, Veh, P = 0.02). **i**-**l** In the VTA, both groups of mice showed similar theta power (**i**: time x group, P = 0.6; time, P = 0.001; group, P = 0.2; **j**: P = 0.8; **k**: P = 0.2; **l**: SCH, P = 0.03, Veh, P = 0.02). **m**-**p** In the dHPC, both groups displayed similar theta power during novelty exposure (**m**: time x group, P = 0.2; time, P = 0.6; group, P = 0.2; **n**: P = 0.3; **o**: P = 0.1; **p**: SCH, P = 0.9, eh, P = 0.8). Inset: magnification of the area enclosed by dashed lines. Two-way RM ANOVA for (**a**, **e**, **i**, **m**). Mann–Whitney test for between group analysis. Wilcoxon signed-rank test for within group analysis. N.S., not significant. *P < 0.05, **P < 0.005. Data are represented as mean ± SEM

**Fig. 7:**
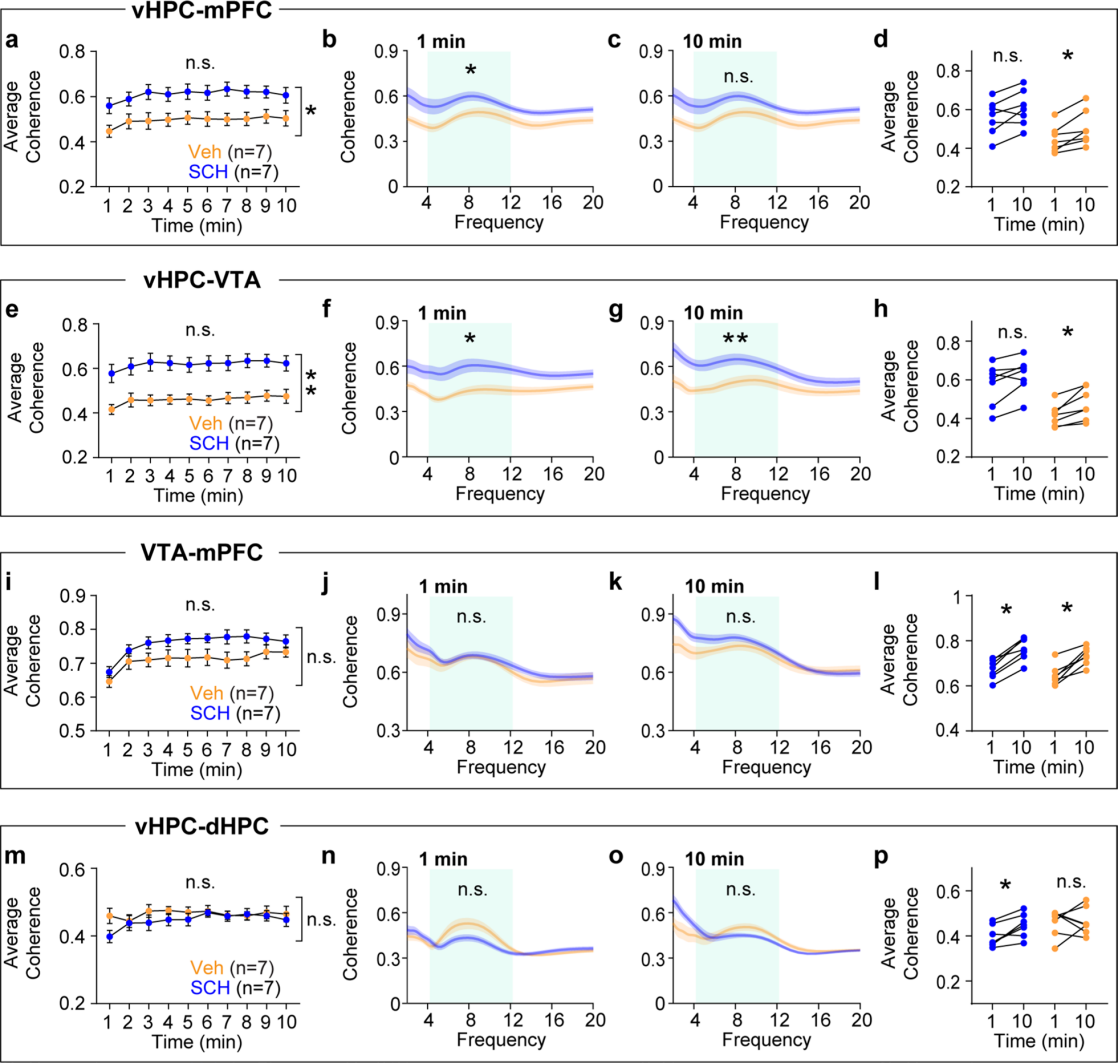
Antagonizing D1 receptors in the vHPC impacts theta coherence dynamics in specific circuits between the HPC, mPFC, and VTA. **a**-**d** The SCH group displayed increased vHPC-mPFC theta coherence compared with the vehicle group. **a**: time x group, P = 0.7; time, P = 0.01; group, P = 0.02; **b**: P = 0.02; **c**: P = 0.07; **d**: SCH, P = 0.08, Veh, P = 0.02). **e**–**h** The SCH group maintained higher vHPC-VTA theta coherence throughout novelty exposure relative to the vehicle group (**e**: time x group, P = 0.9; time, P = 0.02; group, P = 0.002; **f**: P = 0.01; **g**: P = 0.004; **h**: SCH, P = 0.04, Veh, P = 0.02). **i**-**p** Both groups of mice exhibited similar theta coherence in the VTA-mPFC (**i**: time x group, P = 0.2; time, P = 0.0002; group, P = 0.08; **j**: P = 0.2; **k**: P = 0.3; **l**: SCH, P = 0.02, Veh, P = 0.02), and vHPC-dHPC (**m**: time x group, P = 0.2; time, P = 0.1; group, P = 0.4; **n**: P = 0.07; **o**: P = 0.7; **p**: SCH, P = 0.03, Veh, P = 0.9). Two-way RM ANOVA for (**a**, **e**, **i**, **m**). Mann–Whitney test for between-group analysis. Wilcoxon signed-rank test for within-group analysis. N.S., not significant. * P < 0.05, ** P < 0.005. Data are represented as mean ± SEM

**Fig. 8:**
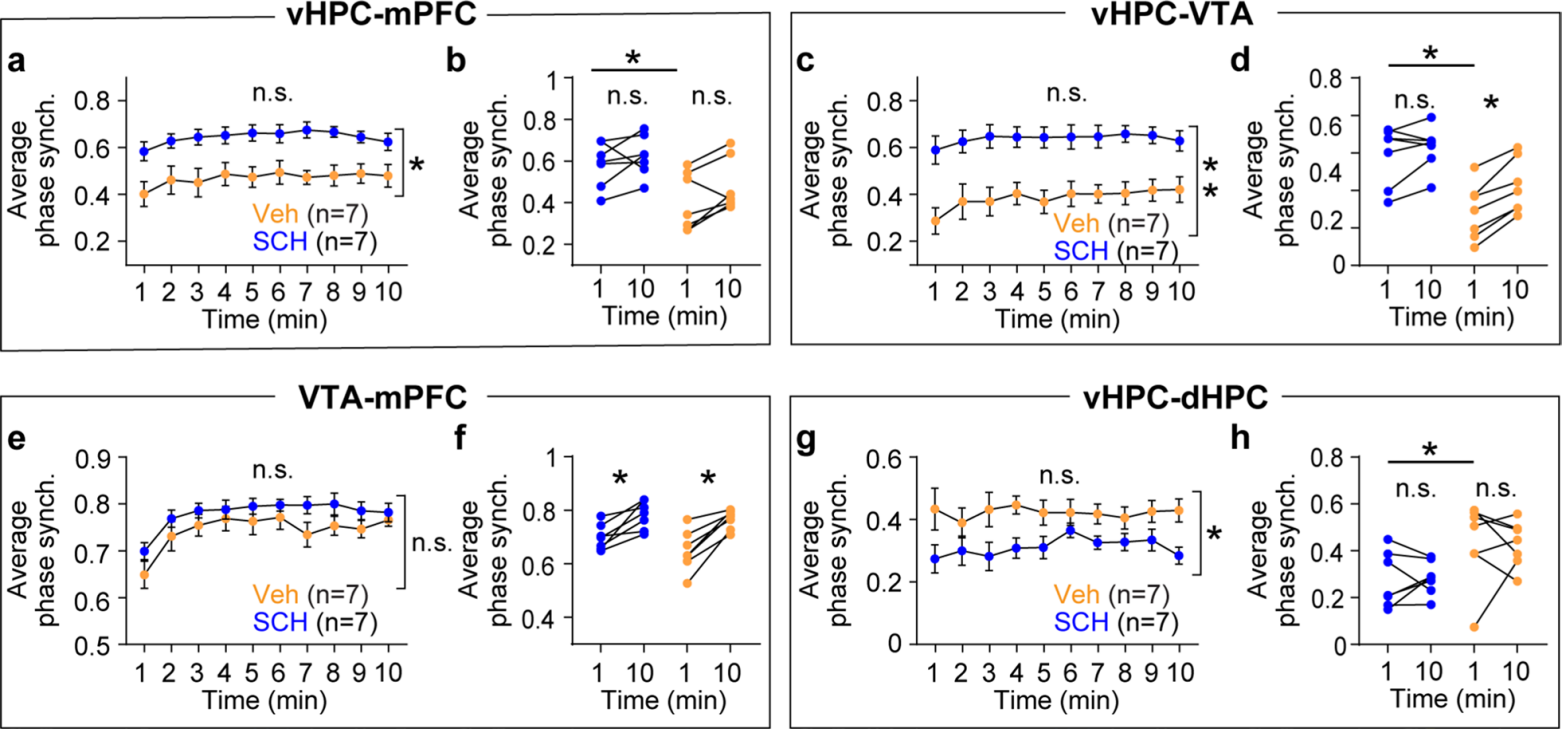
Antagonizing D1 receptors in the vHPC impacts theta phase synchrony dynamics in specific circuits between the HPC, mPFC, and VTA. **a-d** The SCH group exhibited increased theta phase synchrony in the vHPC-mPFC, and vHPC-VTA circuits during novelty exposure compared with the vehicle group (**a**: time x group, P = 0.9; time, P = 0.06; group, P = 0.007; **b**: SCH vs. Veh at 1 min: P = 0.03; SCH vs. Veh at 10 min: P = 0.1; SCH 1- vs. 10min: P = 0.2; Veh 1- vs. 10min: P = 0.1; **c**: time x group, P = 0.3; time, P = 0.03; group, P = 0.003; **d**: SCH vs. Veh at 1 min: P = 0.005; SCH vs. Veh at 10 min: P = 0.07; SCH 1- vs. 10min: P = 0.3; Veh 1- vs. 10min: P = 0.02). **e** &** f** Both groups of mice displayed similar VTA-mPFC theta phase synchrony (**e**: time x group, P = 0.8; time, P = 0.0004; group, P = 0.2; **f**: SCH vs. Veh at 1 min: P = 0.8; SCH vs. Veh at 10 min: P = 0.9; SCH 1- vs. 10min: P = 0.02; Veh 1- vs. 10min: P = 0.02). **g** &** h** The SCH group showed decreased vHPA-dHPC theta phase synchrony (**g**: time x group, P = 0.5; time, P = 0.5; group, P = 0.03; **h**: SCH vs. Veh at 1 min: P = 0.03; SCH vs. Veh at 10 min: P = 0.1; SCH 1- vs. 10min: P = 0.8; Veh 1- vs. 10min: P = 0.7). Two-way RM ANOVA for (**a**, **c**, **e**, **g**). Kruskal–Wallis test followed by Dunn’s post-hoc test for between-group analysis, and Wilcoxon signed-rank test for within-group analysis. N.S., not significant. *P < 0.05, **P < 0.005. Data are represented as mean ± SEM

Novelty stimulates VTA dopamine neurons that project to the vHPC and activates vHPC neurons expressing dopamine D1Rs [5, 8, 14]. Moreover, prior novelty exposure enhances learning and allows learning-dependent increases in theta coherence in the vHPC-mPFC and vHPC-VTA circuits during task training, and these effects are abolished by blocking D1Rs in the vHPC [8, 9]. However, the mechanism by which D1Rs act on behavioral and physiological adaptation to novelty exposure per se is unknown. I therefore examined how blocking D1Rs in the vHPC affects behavior and oscillatory dynamics in the vHPC-mPFC-VTA circuit in response to novelty exposure. Mice were bilaterally infused with the D1R antagonist SCH23390 (SCH) or vehicle into the vHPC twenty minutes before novelty exposure (Fig. 5a). SCH treatment did not affect behavioral parameters, including speed, angular velocity, acceleration, deceleration, and time spent in the center of the arena, during novel arena exploration compared with vehicle treatment (Fig. 5b-f). Physiologically, both groups showed progressive increases in theta power during novelty exposure in the mPFC, vHPC, and VTA as seen in mice exposed to novelty (Fig. 2a-i). However, SCH treatment selectively reduced overall theta power in the vHPC (Fig. 6a-l). The dHPC displayed similar theta power between the two groups (Fig. 6m-p). SCH treatment did not have clear effects on beta- and gamma power in these areas (Supplemental Fig. 3 & 4). Additionally, both coherence and phase synchrony measures indicated that treating SCH into the vHPC resulted in sustained high levels of theta synchrony selectively in the vHPC-mPFC and vHPC-VTA circuits, abolishing gradual increases in theta synchrony (Fig. 7 & 8). Thus, antagonizing D1Rs in the vHPC alters theta dynamics within the vHPC-mPFC and vHPC-VTA circuits without affecting behavior during novelty exposure.

## Discussion

This study unveils how novelty exposure impacts online neuro-oscillatory dynamics in the major learning and memory circuit composed of the mPFC, HPC, and VTA. Distinct from past research on how prior novelty facilitates subsequent memory acquisition, this work revealed characteristic local- and circuit-level theta frequency dynamics that occur over the course of novelty exposure. Although overall neurophysiological signatures of adaptation to novelty are not simply dictated by locomotive activity, D1Rs in the vHPC are critical for novelty-induced dynamics in vHPC-mPFC and vHPC-VTA circuits. Thus, the present study unveiled a latent dynamic state of the brain triggered by novelty.

This work showed that theta power progressively increases in the mPFC, VTA, and most prominently in the vHPC during novelty exposure. Because initially increased fMRI BOLD signals in these areas dissipate as novel stimuli become familiarized [2, 4] and novelty increases overall theta power in the mPFC and vHPC [2, 4, 8], I hypothesized that theta power would initially increase and then decrease over the course of novelty exposure. The observed phenomena mismatch both the hypothesis and behavior where increased exploratory behavior subsides as mice become familiarized with the novel arena. This suggests that novelty induces neurophysiological states in the mPFC, vHPC, and VTA, which are not readily accessible through behavior. It has been shown that cells with high excitability likely become engram cells that encode memory [19, 20]. It is plausible that neurons synchronized with increased theta oscillations can become engram cells thereby enhancing memory performance in tasks following novelty. Indeed, vHPC neurons synchronized with augmented theta oscillations during novelty exposure display higher firing rate, and mPFC neurons synchronized with vHPC theta oscillations encode relevant information following novelty [8]. Thus, these findings suggest that novelty-induced increases in theta activity may tune the capability of the memory circuit composed of the mPFC, vHPC, and VTA for effective memory processing. Longitudinal tracking of neuronal activities using calcium imaging combined with LFP recordings will confirm this speculation.

I expected that theta frequency synchrony between the mPFC, vHPC, and VTA would increase during novelty exposure because all of these areas show increasing theta power at similar kinetics. To test this, I computed LFP coherence and phase synchrony. Although novelty induces progressive increases in theta synchrony in the vHPC-mPFC, vHPC-VTA, and VTA-mPFC circuits as expected, phase synchrony measures indicate that this effect is due to an initial weakening of connectivity at the onset of novelty exposure. In other words, theta synchrony in these circuits markedly drops and then recovers to the level observed in the familiar group over the course of novelty exposure. This initial weakening of synchrony can be due to the reorganization of neuronal spiking at particular phases of theta oscillations [8, 21]. The time-dependent changes in theta synchrony mirror the time course of behavior where novelty increases initial exploration, which then normalizes to the level seen in the familiar group. Thus, unlike theta power, theta synchrony dynamics between the mPFC, vHPC, and VTA reflect the processing of information through novelty exposure. Future studies using simultaneous multi-regional neuronal activity recordings will further dissect information processing in these circuits through novelty exposure and reveal how theta synchrony dynamics triggered by novelty affect subsequent behavior. Moreover, it remains to be determined whether other variables such as location, head direction, or breathing affect these findings [22–24].

The present study demonstrated that antagonizing D1Rs in the vHPC selectively reduces overall theta power in the vHPC and strengthens theta synchrony in the vHPC-mPFC and vHPC-VTA circuits throughout novelty exposure, without affecting behavior. This is in line with previous reports that blocking D1Rs in the vHPC abolishes the enhancement of memory acquisition following novelty by dampening vHPC theta power and strengthening theta synchrony in the vHPC-mPFC and vHPC-VTA circuits [8, 9]. The latter effect prevents learning-dependent increases in theta synchrony in these circuits [9]. Similarly, this study found that the strengthened theta synchrony by D1R blockage prevents further strengthening over the course of novelty exposure. These findings confirm that D1Rs in the vHPC play a central role in novelty processing within the vHPC-mPFC-VTA circuit, without influencing locomotive behavior. It should be noted, however, that pharmacological blockage of D1Rs lacks temporal specificity, and the exact time window in which D1Rs mediate novelty processing remains to be determined. Dissecting how dopaminergic dynamics modulate this circuit for novelty processing will broaden our understanding of the impact of novelty on cognition.

It is worth noting that I did not find any impact of novelty on the dHPC. This is surprising because infusing the D1R antagonist SCH23390 into the dHPC impaired the enhancement of memory retention following novelty exposure [25, 26]. Moreover, the lack of D1Rs in dHPC place cells diminishes their ability to detect spatial novelty [27]. Given that the vHPC provides novelty information to the dHPC [28], it is plausible that the vHPC is the primary region for novelty processing while the dHPC plays a secondary role. Further studies on dopaminergic innervation over the dorsoventral axis of the hippocampus will be crucial to confirm this notion.

I discovered that novelty induces a distinct pattern of theta oscillatory dynamics in specific brain circuits, which could enhance subsequent cognitive function. Because novelty processing is compromised in many neurodevelopmental and neuropsychiatric disorders such as autism and schizophrenia, the insights gained from this research can help develop strategies for targeted modulation of theta oscillatory dynamics to treat cognitive dysfunctions in these conditions.

## Methods

This study used a subset of data collected for previously published works [8, 9]. Specifically, data from the mice implanted with electrodes in the mPFC, vHPC, and VTA (see Drive implant) were selected to examine the simultaneous interactions between these areas. Unlike the previously published reports demonstrating how novelty exposure enhances subsequent learning [8, 9], the present study focused on the time course of neural dynamics in the mPFC-vHPC-VTA circuit through novelty exposure per se.

### Subjects

Three-month-old male C57BL/6J mice (Jackson Labs) were kept on a 12-h light/ 12-h dark cycle with lights on at 7 am. Food and water were available ad libitum. After chronic implant surgery, mice were paired and separately housed in cages divided by a perforated plastic divider to prevent social isolation and protect the implants. Mice were randomly assigned to experimental groups on the day of the experiment. All procedures adhered to the NIH Guidelines and were approved by Columbia University and the New York State Psychiatric Institute Institutional Animal Care and Use Committees (IACUC).

### Surgical procedures

Anesthesia was induced at 2% and maintained at 0.8% isoflurane throughout surgery. Mice were placed on a heating pad. Carprofen (0.15 mL) and dexamethasone (0.05 mL) were administered subcutaneously before the surgery. Experiments were performed four weeks after surgery when the mice were fully recovered.

### Drive implant

A tungsten wire field electrode (76 μm diameter) was implanted into the dHPC (targeting CA1 pyramidal layer; 1.9 mm posterior to, 1.3 mm lateral to, 1.26 mm below bregma), vHPC (targeting ventral CA1/subiculum; 3.2 mm posterior to, 3.3 mm lateral to, 4.59 mm below bregma), and VTA (3.2 mm posterior to, 0.32 mm lateral to, 4.43 mm below bregma). A bundle of 13 tungsten wire stereotrodes (13 μm diameter) was implanted into the mPFC (targeting prelimbic/ infralimbic cortex, layer II/III;1.8 mm anterior to, 0.3 mm lateral to, 2.1 mm below bregma). The wires were connected to a 36-channel electrode interface board (EIB, Neuralynx), which was fixed to the skull with dental cement. Reference and ground screws were placed in the skull overlying the frontal cortex and cerebellum, respectively and fixed with dental cement. The wires attached to the screws were connected to the EIB.

### Cannula implant

Guide cannulae (26 gauge; Plastics One, Roanoke, VA 24018) were implanted bilaterally into the vHPC at a 10-degree angle (3.2 mm posterior to, 3.88 mm lateral to, 3.3 mm below bregma) and secured with dental cement. The dummy cannulae (Plastics One) were inserted into the guide cannulae until the day of drug infusion. A tungsten wire field electrode was attached to a guide cannula with the tip of the electrode positioned 0.7 mm below the cannula. Additional field electrodes were implanted into the dHPC and VTA, and a stereotrode bundle was implanted into the mPFC as described above. On the day of infusion, 33-gauge internal cannulae with a 0.5 mm projection were inserted into the guide cannulae.

### Histology

After completing behavioral experiments, electrode placements were confirmed by visual inspection of electrolytic lesions. Mice were anesthetized using a ketamine/xylazine mixture, and lesions were created by passing a 50 μA current to an electrode for 20 s. Mice then underwent transcardial perfusion with PBS followed by 4% paraformaldehyde in PBS. Brains were fixed overnight at 4°C in 4% paraformaldehyde and cryoprotected in 30% phosphate-buffered sucrose at 4°C for three days. Using a cryostat, brains were sectioned into 40 μm slices and mounted with DAPI Fluoromount-G mounting medium (Southern Biotech, Cat. #: 0100–20). Only data from validated recording sites were included in data analyses.

### Drug

The selective D1-like antagonist SCH23390 (Tocris, Cat. #: 0925) was prepared as a 100 mM stock solution in saline and delivered at 1 mM final concentration in saline.

### Behavior

Previous reports indicate that exposing mice to a brightly lit open arena induces anxiety-related behavior and increases vHPC-mPFC theta synchrony. Notably, these effects were not observed when the same experiments were performed in the dark [29]. Moreover, introducing bright light during novel arena exposure abolishes novelty-induced synaptic depression [30]. Therefore, all experiments were performed in the dark to avoid any non-specific anxiety-related effects on brain circuitry.

Four weeks post-surgery, food restriction began to maintain 85% of the pre-restriction weight until the end of the experiment. On the same day, a three-minute gentle handling was started and continued for three days to acclimate mice to the experimenter. Then, for the next three days, a subset of mice (the familiar group) was exposed to a circular arena (50 cm in diameter and 25 cm in height) for 10 min to allow them to be familiarized with the arena. The rest of the mice (the novel group, the cage mates of the familiar group) remained in their home cage. Finally, the day after the last day of arena exposure of the familiar group, both groups of mice were exposed to the same arena for 10 min.

### D1 receptor antagonist experiment

Mice were habituated to cannulation procedures for five days prior to the day of infusion to minimize potential novelty associated with the procedures. SCH23390 (100 nl, 1 mM) or vehicle (saline, 100 nl) was loaded into a 10 μL Hamilton syringe and administered bilaterally to the vHPC at 50 nl /min using a Harvard Apparatus Pump II Dual Syringe micropump. Following administration, the injection cannulae remained in place for five minutes to let the injected fluid diffuse. After twenty minutes in homecage, mice were introduced to the novel circular arena. Of note, although injecting 3.1 mM of SCH into the dHPC suppresses novelty-enhanced memory consolidation [8], injecting such a high concentration into the vHPC leads to severe sedation or bradykinesia for several hours. The injected 100 nl of 1 mM SCH23390 is equivalent to 0.1 µmol/kg, which is the minimum effective concentration that acts on D1 receptors [31]. SCH23390 is also an agonist for 5-HT2 receptors, but the minimum effective concentration for these receptors is 30 µmol/kg [31]. Therefore, although it cannot be ruled out, it is unlikely that the observed effect of SCH23390 treatment is due to the activation of 5-HT2 receptors.

### Neural data analysis

Neurophysiological recordings were conducted while mice were exploring the circular arena. A Digital Lynx system (Neuralynx, Bozeman, MT) was used to amplify, band-pass filter (1–1000 Hz) and digitize the electrode recordings. LFPs were sampled at 2 kHz. In order to mitigate the effect of animal movement [32], recordings were analyzed when mice were moving (6–30 cm/s). This range was determined based on the observed bimodal distribution of speeds during the exposure [8]. All analyses were performed when both groups of mice were moving within the same range of speed and acceleration. Data were analyzed using custom-written MATLAB scripts. Raw LFP data were normalized to the root mean square of the voltage signal for the entire session to address impedance variability across electrodes. Power and coherence were calculated using the wavelet method (MATLAB wavelet toolbox). Phase information was extracted using the Hilbert transform to obtain theta phase angles. Phase angle differences between two LFP signals were calculated as follows:

$$Phase\,synchrony = \left| {n^{ - 1} \,\sum\limits_{t = 1}^{n} {e^{{j\left( {\phi_{xt} - \phi_{yt} } \right)}} } } \right|$$

n is the number of time points. Pi is a phase angle from brain area x or y at time t.

### Statistics

Graphpad Prism 9 was used for statistical analysis. All statistical tests were two-tailed. Differences were considered statistically significant when P < 0.05. All data passed normality tests (Anderson–Darling and Shapiro–Wilk), allowing the use of two-way RM ANOVA tests. Non-parametric tests were also used for within- and between-group analyses of LFP data.

## Supplementary Information

Additional file 1

## Data Availability

All data are available from the corresponding author upon reasonable request.

## Abbreviations

LFP: Local field potential
mPFC: Medial prefrontal cortex
vHPC: Ventral hippomcapus
dHPC: Dorsal hippocampus
VTA: Ventral tegmental area
D1R: D1 receptor
