# Supplementary material for: Novelty triggers time-dependent theta oscillatory dynamics in cortical-hippocampal-midbrain circuitry
Source: Mol Brain. 2024 Dec 18;17:94. doi: 10.1186/s13041-024-01167-6 (PMC11654259; doi:10.1186/s13041-024-01167-6)
Supplement: Supplementary file 1 — Additional file 1 [file 13041_2024_1167_MOESM1_ESM.docx]

**
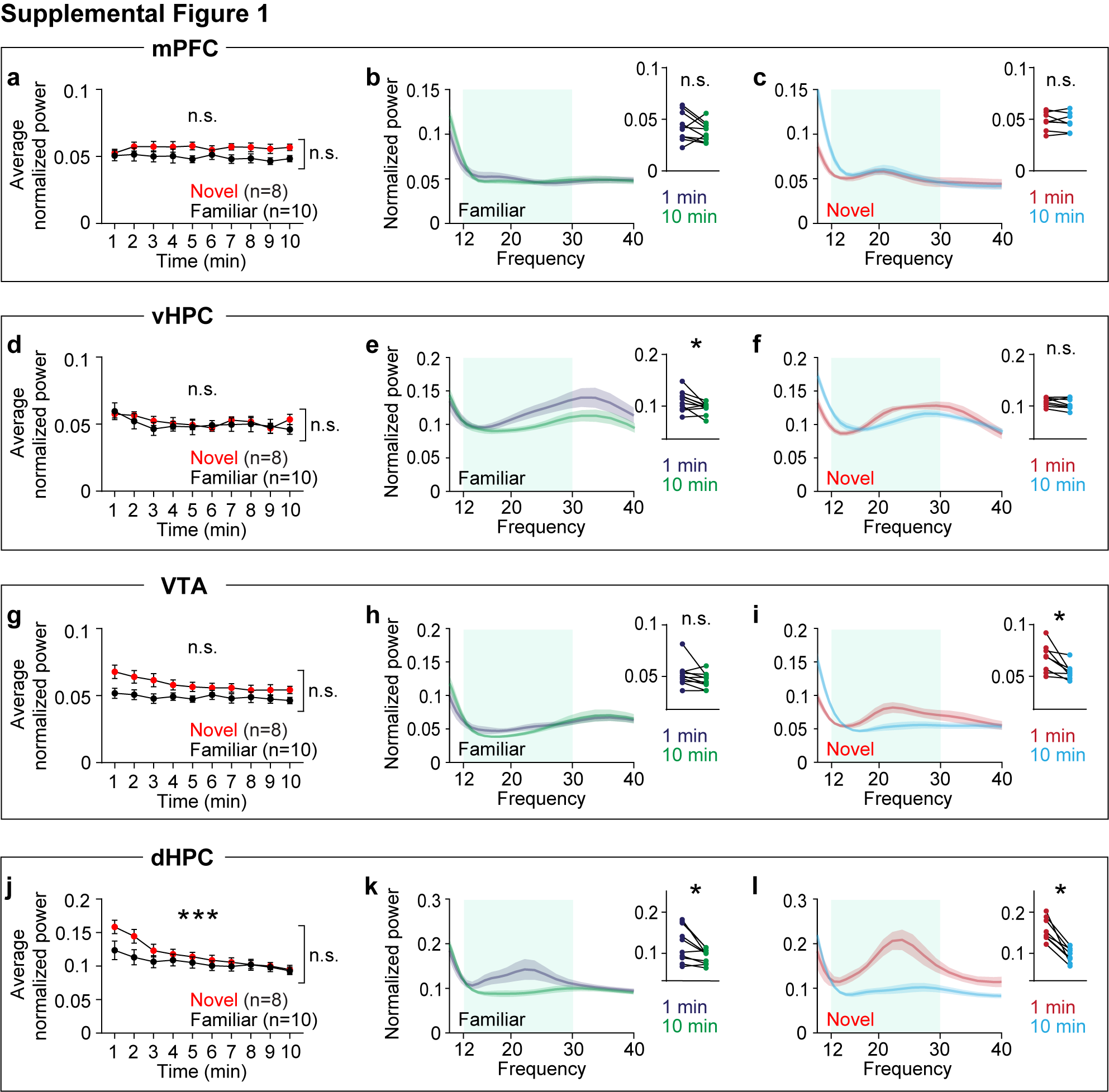
Supplemental Figure 1**. Beta power dynamics in the HPC-mPFC-VTA circuit during arena exposure. (**a**-c) In the mPFC, both groups of mice displayed similar beta power changes over 10 minutes of arena exposure (**a**: time x group, P = 0.5; time, P = 0.5; group, P = 0.1; **b**: P = 0.5; **c**: P = 0.05). (**d**-**f**) In the vHPC, both groups of mice displayed similar beta power changes over 10 minutes of arena exposure (**d**: time x group, P = 0.5; time, P = 0.04; group, P = 0.7; **f**: P = 0.1). Within-group analysis between 1- and 10 min revealed decreasing beta power in the familiar group (**e**: P = 0.04). (**g**-**i**) In the VTA, both groups of mice displayed similar beta power changes over 10 minutes of arena exposure (**g**: time x group, P = 0.07; time, P = 0.03; group, P = 0.05; **h**: P = 0.06). Within-group analysis between 1- and 10 min revealed decreasing beta power in the novel group (**i**: P = 0.008). (**j**-**l**) In the dHPC, both groups displayed decreasing beta power during arena exposure with the novel group exhibiting a bigger effect (**j**: time x group, P < 0.0001; time, P < 0.0001; group, P = 0.3; **k**: P = 0.01; **l**: P = 0.008). Inset: average normalized power in the shaded area. Two-way RM ANOVA for (**a**, **d**, **g**, **j**). Wilcoxon signed-rank test for the rest. N.S., not significant. * P < 0.05, *** P < 0.0005. Data are represented as mean ± SEM.

**
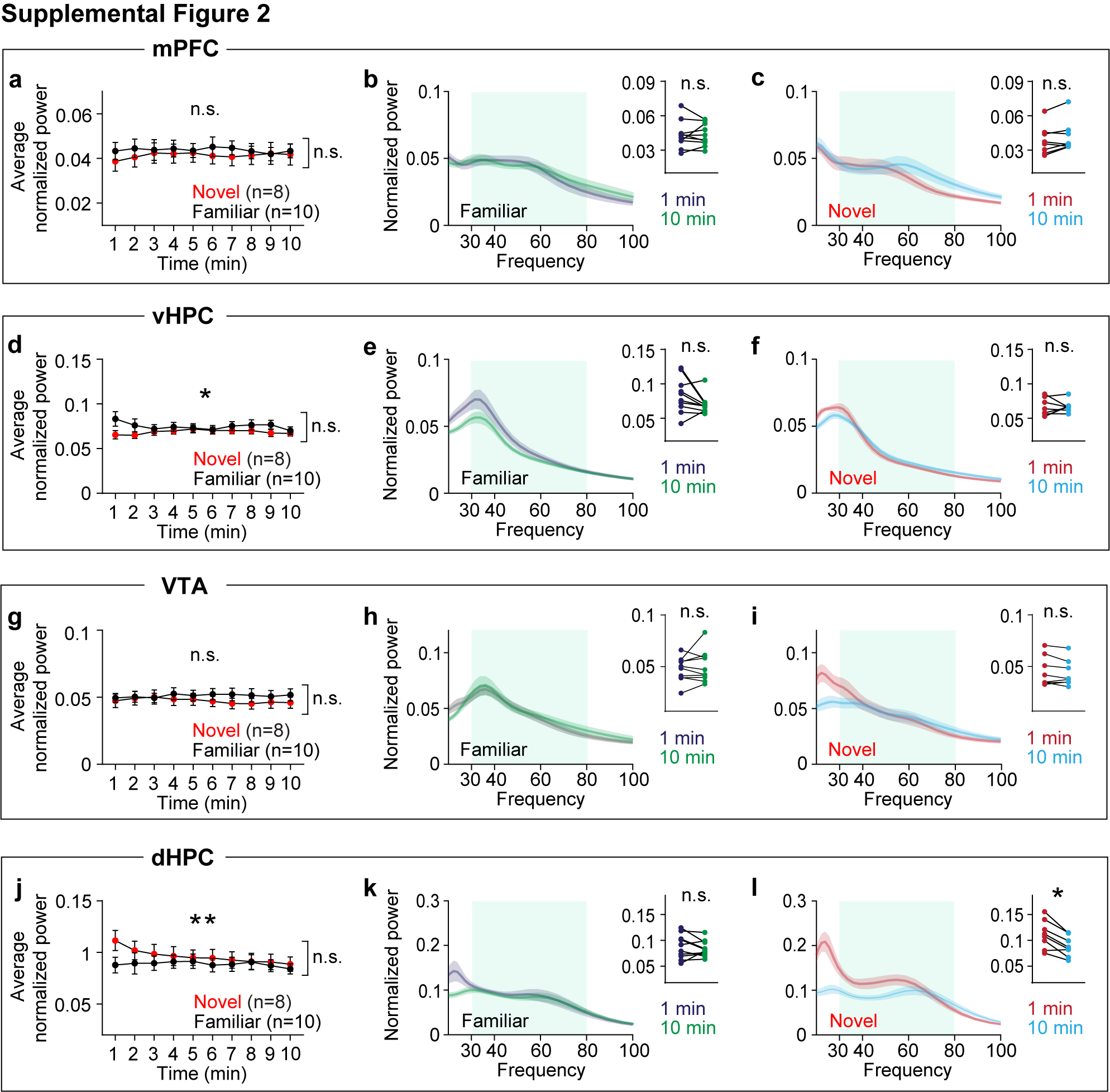
Supplemental Figure 2**. Gamma power dynamics in the HPC-mPFC-VTA circuit during arena exposure. (**a**-**i**) Both groups of mice displayed similar gamma power changes over 10 minutes of arena exposure in the mPFC (**a**: time x group, P = 0.6; time, P = 0.5; group, P = 0.7; **b**: P = 0.8; **c**: P = 0.1), vHPC (**d**: time x group, P = 0.02; time, P = 0.4; group, P = 0.3; **e**: P = 0.1; **f**: P = 0.7), and VTA (**g**: time x group, P = 0.2; time, P = 0.5; group, P = 0.5; **h**: P = 0.8; **i**: P = 0.5). (**j**-**l**) In the dHPC, the novel group exhibited progressive decreases in gamma power compared with the familiar group (**j**: time x group, P = 0.0006; time, P = 0.05; group, P = 0.5; **k**: P = 0.6; **l**: P = 0.02). Inset: average normalized power in the shaded area. Two-way RM ANOVA for (**a**, **d**, **g**, **j**). Wilcoxon signed-rank test for the rest. N.S., not significant. * P < 0.05, ** P < 0.005. Data are represented as mean ± SEM.

**
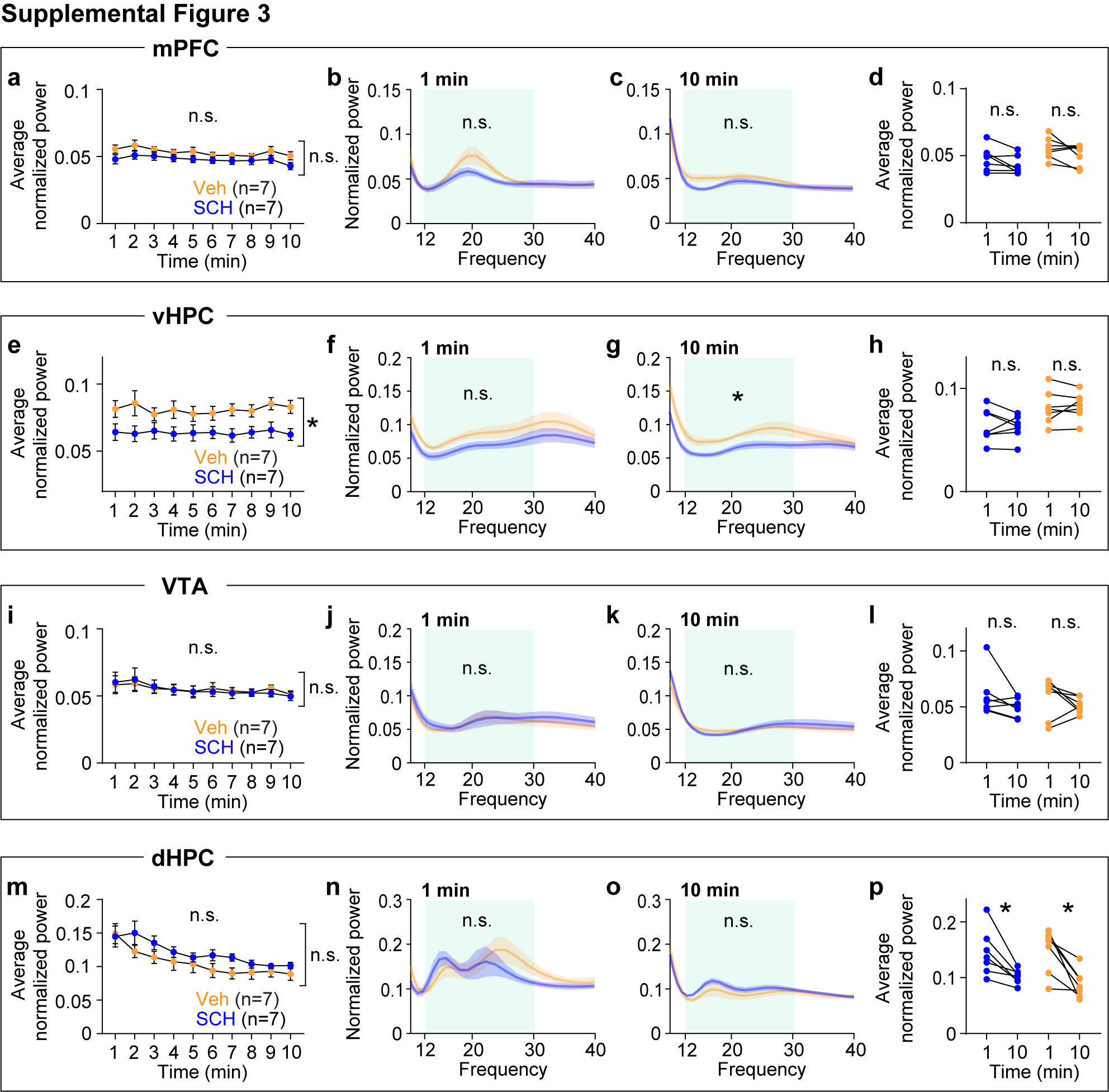
Supplemental Figure 3**. The effect of D1 receptor antagonism in the vHPC on beta power dynamics in the HPC-mPFC-VTA circuit during novelty exposure. (**a**-**d**) In the mPFC, both groups of mice displayed similar beta power (**a**: time x group, P = 0.7; time, P = 0.03; group, P = 0.1; **b**: P = 0.1; **c**: P = 0.1; **d**: SCH, P = 0.08, Veh, P = 0.2). (**e**-**h**) In the vHPC, the SCH group exhibited decreased beta power relative to the Veh group (**e**: time x group, P = 0.5; time, P = 0.4; group, P = 0.03; **f**: P = 0.07; **g**: P = 0.01; **h**: SCH, P = 0.8, Veh, P = 0.9). (**i**-**l**) In the VTA, both groups of mice displayed similar beta power (**i**: time x group, P = 0.9; time, P = 0.1; group, P = 0.9; **j**: P = 0.5; **k**: P = 0.9; **l**: SCH, P = 0.08, Veh, P = 0.4). (**m**-**p**) In the dHPC, both groups displayed decreasing beta power during novelty exposure (**m**: time x group, P = 0.2; time, P < 0.0001; group, P = 0.2; **n**: P = 0.6; **o**: P = 0.2; **p**: SCH, P = 0.02, Veh, P = 0.02). Two-way RM ANOVA for (**a**, **e**, **i**, **m**). Mann-Whitney test for between-group analysis. Wilcoxon signed-rank test for within-group analysis. N.S., not significant. * P < 0.05. Data are represented as mean ± SEM.

**
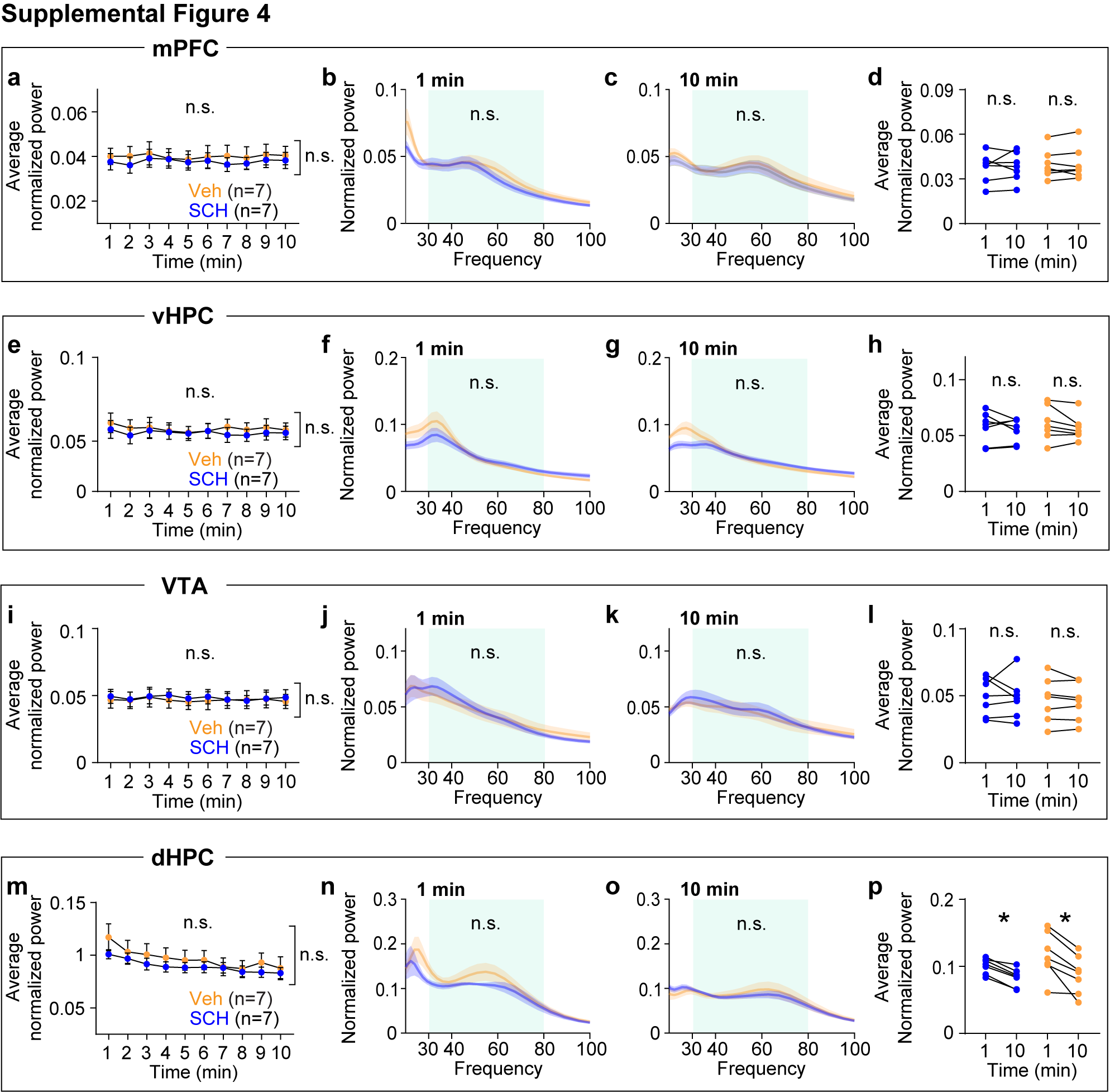
Supplemental Figure 4**. The effect of D1 receptor antagonism in the vHPC on gamma power dynamics in the HPC-mPFC-VTA circuit during novelty exposure. (**a**-**l**) Mice treated with SCH or vehicle displayed similar gamma power during novelty exposure in the mPFC (**a**: time x group, P = 0.8; time, P = 0.3; group, P = 0.7; **b**: P = 0.9; **c**: P = 0.9; **d**: SCH, P = 0.9, Veh, P = 0.8), vHPC (**e**: time x group, P = 0.7; time, P = 0.3; group, P = 0.7; **f**: P = 0.7; **g**: P = 0.9; **h**: SCH, P = 0.7, Veh, P = 0.2), and VTA (**i**: time x group, P = 0.8; time, P = 0.4; group, P = 0.8; **j**: P = 0.8; **k**: P = 0.8; **l**: SCH, P = 0.9, Veh, P = 0.5). (**m**-**p**) In the dHPC, both groups exhibited progressive decreases in gamma power (**m**: time x group, P = 0.1; time, P = 0.0005; group, P = 0.5; **n**: P = 0.3; **o**: P = 0.8; **p**: SCH, P = 0.02, Veh, P = 0.02). Two-way RM ANOVA for (**a**, **e**, **i**, **m**). Mann-Whitney test for between-group analysis. Wilcoxon signed-rank test for within-group analysis. N.S., not significant. * P < 0.05. Data are represented as mean ± SEM.
